# Toward Impactful Collaborations on Computing and Mental Health

**DOI:** 10.2196/jmir.9021

**Published:** 2018-02-09

**Authors:** Rafael Alejandro Calvo, Karthik Dinakar, Rosalind Picard, Helen Christensen, John Torous

**Affiliations:** ^1^ Wellbeing Supportive Technology Lab School of Electrical and Information Engineering University of Sydney Sydney Australia; ^2^ Massachusetts Institute of Technology Media Lab Cambridge, MA United States; ^3^ Black Dog Institute Sydney Australia; ^4^ Division of Clinical Informatics, Department of Medicine Beth Israel Deaconess Medical Center Harvard Medical School Boston, MA United States; ^5^ Department of Psychiatry Beth Israel Deaconess Medical Center Harvard Medical School Boston, MA United States

**Keywords:** mental health, human-computer interaction, digital interventions, interdisciplinary collaboration

## Abstract

We describe an initiative to bring mental health researchers, computer scientists, human-computer interaction researchers, and other communities together to address the challenges of the global mental ill health epidemic. Two face-to-face events and one special issue of the *Journal of Medical Internet Research* were organized. The works presented in these events and publication reflect key state-of-the-art research in this interdisciplinary collaboration. We summarize the special issue articles and contextualize them to present a picture of the most recent research. In addition, we describe a series of collaborative activities held during the second symposium and where the community identified 5 challenges and their possible solutions.

## Introduction: Computing and Mental Health

Whether measured in terms of suffering, disability, or economics, the devastating impact of mental ill health continues to grow. In 2017, the World Health Organization labeled depression as the single leading cause of global disability [1], and in 2016 there were nearly 800,000 suicides worldwide [2]. With a projected global shortage of mental health clinicians, especially in low- and middle-income countries [3], new solutions are urgently needed. The uniting of computing and mental health fields offers the potential to bring easily accessible, scalable, affordable, and innovative tools for preventing mental health problems and for improving the overall mental health of the global population [4] as the articles in this special issue exemplify. However, the first step toward joint computing and mental health solutions is to actually bring these two often disparate communities together.

**Figure 1:**
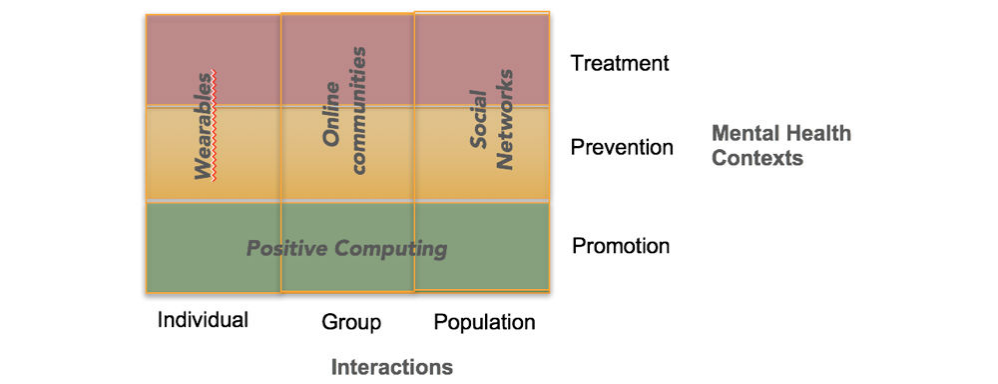
Taxonomy for technologies in mental health and well-being (adapted from Calvo et al [5], with permission from the author).

This joint community started as “a gathering of researchers from health, psychology, psychiatry, and human-computer interaction (HCI) meeting together to consider ways technology can be used to improve psychological wellbeing” (pg 3439) [5]. At the first gathering in 2016, we introduced a schematic and taxonomy to conceptualize technologies to support mental health and well-being (see Figure 1). In Figure 1, the vertical axis represents 3 commonly used categories in mental health care: treatment (interventions aimed at addressing illness), prevention (aiming to reduce risk), and promotion (fostering optimal mental health universally in line with the concept of mental health flourishing [6]). The horizontal axis indicates the target level of the intervention: individual (eg, one-to-one counseling session, journal writing), group (eg, online community intervention), or general population (eg, Facebook). 

Positive computing is defined as “the design and development of technology to support psychological well-being and human potential” [7]. While every taxonomy has limitations, this organizational structure for mental health technologies enables identification of well-researched areas, as well as those deserving of further investigation.

For the inaugural symposium held in Silicon Valley, California, USA, in May 2016, we received 72 submissions, reflecting a high level of interest in the topic. Most articles (n=47, 65%) focused on depression and anxiety. Interestingly, although the HCI community is paying increased attention to ways in which technology can support well-being and health promotion, only 22 (30%) of the total submissions to the first event addressed some aspect of mental health promotion or flourishing. Those submissions categorized as mental health promotion addressed physical health (n=10, 20%), sleep (n=8, 16%), strength (n=12, 24%), psychoeducation (n=14, 28%), and antistigma (n=6, 12%). This focus on illnesses versus health promotion and flourishing may reflect the representation of mental health researchers at the event. During the first event, participants expressed their interest in a follow up event and a special issue. These are described in the following sections.

The second Symposium on Computing and Mental Health was held in May 2017 [8]. It was organized in tandem with a special issue published by JMIR Publications. The symposium format allowed participants to meet face-to-face, discuss issues of significance to the community, and disseminate their work. This meeting was complemented by the special issue, which provided an easily accessible and archival publication of relevant peer-reviewed papers, while also maintaining the open access element that many participants thought was important for multidisciplinary collaborations. Participants with a paper accepted in this special issue were invited to give an oral presentation about their work, while other participants were invited to present a poster. In addition, some participants used the event as an educational or networking opportunity.

Further information about the symposiums and special issue can be found at the Computing and Mental Health website [9], and the call for papers for this special issue can be found at the JMIR website [10].

## The Special Issue

The second workshop and associated special issue received 65 submissions, of which 20 were accepted for publication in the special issue. Here we use the taxonomy described in Figure 1 to guide discussion of these articles. Numerous articles actually addressed all 3 mental health contexts of the taxonomy (promotion, prevention, and treatment) but, to better organize this special issue, we have classified articles into a single context.

### Promotion

Promotion, the support of positive mental health, focuses on strategies to improve quality of life rather than the reduction of symptoms. Thus, promotion, in line with the concept of mental health flourishing, aims at preventing future mental health problems and reducing the global burden of mental health. The HCI community has a fast-growing interest in how technologies can be designed to support positive mental health and flourishing [7]. Articles in this category of the special issue, and several other presented as posters, focused on how computing and mental health can be successfully combined to augment mental health and well-being.

Yarosh and Schueller [11] demonstrated how promoting mental health and wellness need not be limited to traditional adult populations. The authors used participatory design approaches to understand children’s (N=12) interpretations of positive psychology concepts. They analyzed almost 500 artifacts produced by the children over the course of 14 participatory design sessions. The article provides insights into how children conceive mental health promotion skills such as gratitude, mindfulness, and problem solving. Peters and colleagues [12] also used participatory design approaches to explore how young people conceive their lives with asthma as a window into mental health promotion and understanding their psychological needs [13]. Participants produced artifacts (collages, paper prototypes, and concept maps) that provided evidence of how important basic psychological needs (eg, autonomy, competence, and relatedness) are for their quality of life. The focus here was an app, a technology to be used by a single individual. Also focusing on an individual device, Zhu and colleagues [14] evaluated a physical device to encourage mental health promotion, a mindful breathing tool (a single-user technology) for stress reduction. The evaluation consisted of a pilot study and structured interviews to learn how this device benefited mental health promotion. Paredes and team [15] explored how the daily commute could be used to improve mental health through mindfulness interventions. Participants described their individual perceptions of movements and vibrotactile patterns in a driving simulation.

Rather than talking directly with potential users, Huang and Bashir [16] explored how information cues, such as ratings and reviews in the app store, may affect mental health promotion through the adoption of mental health apps. Their results show that ratings and reviews have higher correlations with installs than price and privacy settings.

Saha and colleagues [17] studied mental health promotion through public awareness about schizophrenia using data from Facebook’s advertising platform. Facebook collects data from within its products (eg, likes) and from websites that use its marketing tools. These data, which are generally used for personalizing ads, were used instead to estimate a schizophrenia awareness index. Finally, Nicholas and colleagues [18] presented results from a survey completed by experts examining what they thought were the key issues with electronic health in psychiatry, including how it could be used for mental health promotion. The study identified 10 issues: access to care, integration and collaboration, education and awareness, mental health stigma, data privacy, trust, understanding and assessment of mental health, government and policy, optimal design, and engagement.

### Prevention and Monitoring

While a focus of the current mental health system is often treatment, prevention and monitoring are critical toward avoiding the need for treatment. For example, referring patients for early treatment can alter the entire course of chronic lifelong illnesses such as schizophrenia and minimize the severity and disability from the disease [19]. The articles discussed below offer innovative tools and approaches to better quantify and understand the lived experience of mental illness in ways that can enable early detection or real-time responses that are difficult for clinical care to offer today.

Haskins and colleagues [20] conducted a randomized trial of a Web app for alcohol screening, brief intervention, and referral to treatment versus a control condition. They found that participants using the Web app were more likely to seek help from an alcohol treatment provider, but the intervention did not lead to reduced risky alcohol use, suggesting the complexity of this issue. Web apps are not the only tools that can be used for prevention and monitoring. For instance, Delgado-Gomez and colleagues [21] used Microsoft Kinect sensors to develop a novel assessment of attention-deficit/hyperactivity disorder. The results suggest an innovative way for using consumer sensors in clinical assessment and how new information, including whole-body movements through space, can be important mental health metrics. Chow and colleagues [22] used the global positioning system sensors available in mobile phones to model the relationship between state affect and the duration of time spent at home. Their findings that depression and anxiety are associated with staying-at-home behavior patterns offer both a new tool to monitor risk and an actionable signal to guide real-time physical activity interventions. Parra and colleagues [23] studied new biometric approaches using a combination of facial expressions, voice recording, heart rate, and electrodermal activity to assess attachment security. The authors found evidence of unique physiological, behavioral, and linguistic markers of attachment security, offering a model of personalized digital biomarkers. Other groups also explored digital biomarkers, with Saeb and colleagues [24] pairing a mobile phone app to capture sleep data with machine learning algorithms that together achieved significant classification accuracy (88% on 10-minute windows). Aledavood and colleagues [25] also presented a novel mobile phone app designed for broad and customizable data capture in mental health. Their article describes the design of a software platform that integrates and manages mental health data from many sources, including mobile phones, Internet of things devices, surveys, actigraphs, and social media content. Social media also offers a novel space for prevention and monitoring, and Mowery and colleagues [26] contributed an innovative scheme for annotating signs and symptoms of depression: a dataset of 9300 tweets originally used in the 2015 Computational Linguistics and Clinical Psychology Shared Task. Park and Conway [27] used computational linguistic approaches to study how emotion-related language changes over time for members of a depression community on the widely used Internet forum Reddit. The diversity of tools and methods in the above articles represents the growing potential to soon offer mental health monitoring and prevention tools that are affordable, scalable, and personalized.

### Clinical Interventions and Treatment

While the most frequent interventions in mental health today include face-to-face therapy or medications, or both, digital interventions are rapidly expanding. Presentations at the conference focused on novel ways to use current technologies to deliver more personalized, convenient, and affordable care.

Boudreaux and colleagues [28] designed, built, and tested a Web-based safety planning intervention. The design followed user-centered practices in a multidisciplinary team. Kaiser and Laireiter [29] described DynAMo, a tool that could help researchers and psychotherapy practitioners in treatment planning and monitoring of process and outcomes. This open source tool provides mechanisms for quantifying, modeling, and visualizing the progress of a patient while completing psychotherapy treatment. Mandryk and Birk [30], Aguilera and colleagues [31], and Hoermann and colleagues [32] focused on direct interactions with patients. Mandryk and Birk [30] described an approach to using games that can engage patients with interventions. Aguilera and colleagues [31] studied the impact of augmenting cognitive behavioral therapy through text messaging in a randomized controlled study among lower-income patients with depression compared with a control group receiving only cognitive behavioral therapy. Their finding of increased engagement with treatment in the text messaging group, though with similar clinical outcomes to those of the control group, suggests the potential of texting as an intervention. Hoermann and colleagues [32] systematically reviewed synchronous text-based dialogue systems. Dialogue systems can be of two types: fully automated, where end users chat with a bot; and augmentation systems, where they chat with a human. This literature review focused on how these systems are being used in mental health intervention.

## Challenges and Solutions: The Research Community’s Perspective

**Figure 2:**
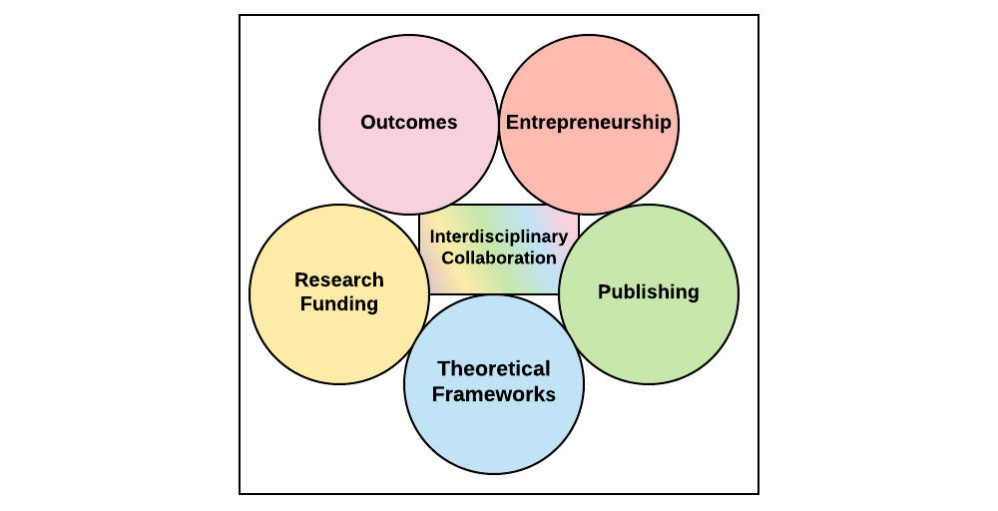
The 5 challenges presented at the symposium and the central role of interdisciplinary collaboration as a solution toward each.

The symposium included several activities aimed at sharing experiences among the participants, discovering common challenges, and discussing possible solutions. In this section, we discuss the 5 themes of challenges discussed. Themes were picked ahead of time from an online poll completed by attendees 2 weeks prior to the symposium. Based on responses, the final themes selected were as follows: entrepreneurship, publishing, funding, theoretical frameworks, and outcomes. At the symposium, attendees separated into 5 groups, 1 group for each theme, with each group comprising approximately 15 members. The groups spent approximately 30 minutes brainstorming a list of challenges related to their theme and then shared their initial results on a large poster. All attendees were given 20 minutes to visit all 5 posters, leave comments, and offer solutions. After this period, each group then spent the next 30 minutes reviewing comments, brainstorming ideas, and listing potential solutions. This list of final solutions was presented and shared with the entire group, and everyone was invited to comment. Here we present only a summary (Figure 2) of those final outcomes based on an analysis of the artifacts and discussions by RAC and JT.

### Challenges and Solutions #1: Entrepreneurship

While within this group there was general agreement on the need for greater academic and industry collaboration, numerous other issues were raised. One was that intellectual property in mental health is “soft,” meaning that it can be difficult to patent or protect innovations in this space. Another discussion point centered on the difficulty in targeting entrepreneurship efforts to the correct audience, which may include governments, insurers, hospital systems, employee benefit programs, end users, and more. While the goal of all stakeholders is improved mental health, the approach, metrics of success, and business models dramatically differ by stakeholder. Another issue raised was that mental health stigma often drives away potential users and investors. Many recognized that creating successful products in this space requires diverse collaborations with mental health professionals, designers, data scientists, end users, and more. Building such teams is feasible but often requires significant effort, organization, and upfront cost. Many mental health professionals noted they might not even know where to start in building such a team or finding the right business partners with whom to connect. The different lexicons and priorities of stakeholders was raised as another set of barriers to forming teams. Finally, the frequent tension between industry incentives for return on investment and profit versus research goals and open dissemination of knowledge was also raised.

Numerous solutions to the entrepreneurship challenge were discussed. There was a general agreement that education about the entrepreneurship process by the mental health and HCI communities is needed. Many noted that they would like to attend workshops and sessions that introduced business concepts and taught the skills needed to navigate the entrepreneurial space. To better align incentives and goals, several suggested that social enterprise ventures are a good target for collaboration between academia and industry. Many also supported the creation of programs that would match clinicians and HCI professionals with potential business partners. Other also suggested that, by using crowdfunding, it might be possible to self-support early-stage efforts and thus bypass the need for entrepreneurship.

All of these above suggestions revolved around means to increase collaboration between the mental health, HCI, and entrepreneur communities.

### Challenges and Solutions #2: Publishing

A second challenge addressed by the group involved publishing. Many noted that, while the academic publication process is often cumbersome and slow, mobile health research evolves rapidly. Waiting up to 6 months for peer review and then longer for actual publication can result in delays of more than 1 year from manuscript submission to published article. For work at the intersection of mental health and HCI, there are often few peer reviewers who can bridge both domains and offer balanced feedback. Many also noted the publication bias for positive results that has created a lack of knowledge regarding how many mobile health studies do not succeed. Others pointed out that many journals, especially HCI-focused ones, favor novelty in submissions and there is less incentive or ability to publish on actual implementation or reproducibility research. Concerns about how to maintain a human participants’ privacy when publishing small case studies or N=1 experiments were also noted as a barrier for publication.

Numerous solutions toward publication challenges were presented. One novel concept was the notion of segmented peer review, with mental health and HCI experts each dividing responsibility for reviewing certain sections of a paper. There was also broad consensus on the need for more interdisciplinary journals that bring together expertise in mental health and HCI. Others suggested the promulgation of guaranteed publication for clinical studies with preapproved methods. While this would require careful screening of study design and approach, such a system would remove bias toward publishing only positive findings. Finally, many agreed on the need for further online prepublication distribution of papers (eg, through arxiv.org), and even the desire to make prepeer-review prints available in order to facilitate dissemination that can keep pace with innovation. Like the entrepreneurship challenge outlined above, new and expanded collaborations were noted as the key to sharing and publishing works at the intersection of HCI and mental health.

### Challenges and Solutions #3: Research Funding

Related to entrepreneurship, the challenge of bringing the necessary funding resources to digital mental health projects was also raised. All agreed that a primary challenge of this space remains a funding gap for mental health works bridging academic disciplines, spanning multiple countries, and even involving multiple industries. Without a primary “home” in academia or industry, dedicated funding is a challenge. Many noted that, even among dedicated academic funding agencies, there are few well-aligned grant mechanisms to support this interdisciplinary work, and that grant-review panels often may not fully understand the unique needs or challenges of the mental health HCI space. Current research funding mechanisms often support a winner-takes-all approach that does not facilitate strong collaborations but instead tends toward supporting isolated efforts by small teams or even individuals. As alluded to above in the entrepreneurship section, a misalignment of incentives, timelines, and values (eg, seeking answers vs outcomes) further complicates the funding landscape. The true nature of many mental health HCI projects is often iterative, but funding for iterative and phased projects is often not available today.

Numerous solutions were described as a way to begin to address funding challenges. Many supported efforts by international centers and foundations that have a broad perspective and willingness to support novel mental health HCI projects. However, realizing that such programs are scarce today, all agreed there is a need for better lobbying for further funding resources from both governments and nonprofits. This could be accomplished by framing mental health as the key challenge of our times and underscoring the potential of this work to aid in recovery, reduce health care costs, and have broad global impact. Many noted that the physics community may serve as a model for building a successful funding message around often abstract and theoretical work. It was suggested the field should unify around a “big message” and present a more coherent message regarding funding priorities. Again, the topic of crowdfunding was raised as a means to provide early support to enable projects to complete pilot assessments. Similarly, all agreed that more education about entrepreneurship and collaboration with industry is necessary. New and stronger collaborations were also seen as the core of all solutions for uniting the right funding with the right opportunities in this space.

### Challenges and Solutions #4: Theoretical Framework

Theory is necessary for hypothesis-driven research, and theory-driven research creates the necessary scaffold for research efforts to support or refute each other. Theories can also provide a framework to understand where studies fit in the larger context and what underpins our understanding of human nature. The current model of collecting numerous digital data streams and then searching for a statistically significant signal is important exploratory and foundational work for the mental health HCI space. The need for theoretical work arises from the fact that models of mental illness such as the biopsychosocial model were developed in an earlier era when the wealth of real-time sensor and survey data offered by the mobile phones of today was unimaginable. For example, new digital mental health data streams offer the potential to help advance the biopsychosocial model by offering a bridge to unite biological and behavioral theories of illness [4]. New and updated models are needed that better account for the novel longitudinal and environmentally aware information streams that current mental health HCI efforts enable. Such a theoretical model should not seek to reduce the understanding of human nature and mental illness to narrow concepts, but rather best use the multivariate nature of available data to increase and incorporate expanding knowledge. This work may thus lead to new digital mental health gold standards and the development of new measurement scales that will replace today’s efforts to correlate digital mental health data to static time-point clinical scales.

Numerous solutions toward creating theoretical frameworks were discussed. One broad solution was to organize further joint conferences and publications between the mental health and HCI communities to encourage cross-pollination of theory and ideas. Another was to carefully search the existing literature for those projects and efforts already bridging the mental health and HCI worlds and review their framing. Others suggested the need to create new scales and outcome measurements for the field, in line with the development of US National Institutes of Health Patient-Reported Outcomes Measurement Information System measures. Another solution was to learn from the pioneering efforts of the Quantified Self community, which has already created its own framework and metrics. Still others noted that, by focusing efforts on solving clinical problems, there is less need to focus on theory, as a problem-focused mindset will naturally produce the desired frameworks. Again, the theme of interdisciplinary collaborations and partnerships was evident at the core of these proposed solutions.

### Challenges and Solutions #5: Outcomes

Along with theory, another challenge considered was outcomes. Many raised the difficulties in establishing causality or casual inferences from predominantly observational studies. Other challenges included the generalizability of outcomes given that many studies are conducted on either proprietary or nonaccessible digital platforms, use statistical analyses that are often too vague to reproduce, and focus on small, nonrepresentative populations. Even if outcomes were well defined, many raised the concern that randomized controlled trials may not be the best method for studies, given that an advantage of digital mental health computing efforts is the ability to study mental illness at the personal level and provide individual assessments and recommendations. Further confounding outcomes, several noted that metrics such as reduction in symptom scores on standardized measurements are often of less utility outside of the clinic and that adaptive or functional outcomes such as employment may matter more. Often-secondary outcomes such as user adherence and engagement become of paramount importance when apps are deployed outside of clinical studies. Finally, the difficulties of any outcomes research in this space is compounded by a myriad of confounding variables, such as participants often receiving new mobile phones, additional clinical visits or attention, or payments for increased participation. Isolating the “active ingredient” or true effect size of digital mental health HCI efforts remains challenging, with reports varying wildly on the actual effect size and impact [33].

In response, the group raised several potential solutions. Many discussed the need for more dynamic randomized controlled trails and moving to more adaptive and iterative study designs. The agile science model proposed by Hekler and colleagues was cited as a viable alternative in which studies are more iterative [33]. All agreed on the need to further study and quantify engagement as a primary outcome and to understand how end users’ experience with the app or digital tool itself affects outcomes. Several also proposed, as noted above, that the field will have to develop new scales and outcome measures instead of seeking to match new digital data streams to traditional clinical scales that were developed in a different era and for use with different data. Along similar lines, it will be necessary to consider not only clinical outcomes that we can easily measure with scales but also real-world and adaptive or functional outcomes such as increased social engagement or cognition. Like for all the above challenges, new collaborations, including those with patient communities, were highlighted as the key to both finding and measuring the right outcomes.

## Conclusions and Future Steps

Interdisciplinary collaborations are a powerful way to solve complex problems and emerged as the core solution in our workshop exploring challenges of entrepreneurship, publishing, funding, theory, and outcomes. Mental health and well-being is undoubtedly one of the most significant complex challenges of our generation, and collaboration, with supportive funding, may present the most important opportunity for progress. We know that our technological environments and tools can advance or improve mental health, as demonstrated in the articles included in this special issue. These promising results, and collaborations between the mental health and HCI communities, rely on development of interdisciplinary research communities that are achievable only through face-to-face and online conversations, collaborative projects, and deep thinking and writing. While the challenges discussed in this editorial are significant, the numerous proposed solutions, interdisciplinary collaborations, and enthusiasm of those partaking in the second Symposium on Computing and Mental Health suggest a bright future for this field. While this one symposium and special issue cannot alone speak for the CHI and mental health fields, we hope it offers a path for others to follow and build upon. The need for better mental health globally is growing and presents a serious challenge that can no longer be met by the mental health community working alone. We urge the communities of CHI and mental health to continue to come together to apply the lessons learned here, while continuing to expand and integrate knowledge and practice to achieve better global mental health.

## Abbreviations

HCI: human-computer interaction
